# Rescue of heavy metal effects on cell physiology of the algal model system *Micrasterias* by divalent ions

**DOI:** 10.1016/j.jplph.2013.10.002

**Published:** 2014-01-15

**Authors:** Stefanie Volland, Elisabeth Bayer, Verena Baumgartner, Ancuela Andosch, Cornelius Lütz, Evelyn Sima, Ursula Lütz-Meindl

**Affiliations:** a Plant Physiology Division, Cell Biology Department, University of Salzburg, Hellbrunnerstraße 34, 5020 Salzburg, Austria; b Institute of Botany, Faculty of Biology, University of Innsbruck, Sternwartestraße 15, 6020 Innsbruck, Austria

**Keywords:** AA, ascorbic acid, Ca, calcium, Cd, cadmium, Cr, chromium, EELS, electron energy loss spectroscopy, ESI, electron spectroscopic imaging, Fe, iron, Fe-EDTA, Fe-ethylenediaminetetraacetic acid, Gd, gadolinium, GSH, glutathione, HPLC, high-performance liquid chromatography, Pb, lead, PS II, photosystem II, ROS, reactive oxygen species, TEM, transmission electron microscopy, SA, salicylic acid, UPLC-MS, ultra performance liquid chromatography–mass spectrometry, Zn, zinc, Amelioration of metal effects, Antioxidants, Green algae, Heavy metals, Ions

## Abstract

Recent studies have shown that metals such as copper, zinc, aluminum, cadmium, chromium, iron and lead cause severe dose-dependent disturbances in growth, morphogenesis, photosynthetic and respiratory activity as well as on ultrastructure and function of organelles in the algal model system *Micrasterias denticulata* (Volland et al., 2011, Volland et al., 2012, Andosch et al., 2012). In the present investigation we focus on amelioration of these adverse effects of cadmium, chromium and lead by supplying the cells with different antioxidants and essential micronutrients to obtain insight into metal uptake mechanisms and subcellular metal targets. This seems particularly interesting as *Micrasterias* is adapted to extremely low-concentrated, oligotrophic conditions in its natural bog environment.

The divalent ions of iron, zinc and calcium were able to diminish the effects of the metals cadmium, chromium and lead on *Micrasterias*. Iron showed most ameliorating effects on cadmium and chromium in short- and long-term treatments and improved cell morphogenesis, ultrastructure, cell division rates and photosynthesis. Analytical transmission electron microscopic (TEM) methods (electron energy loss spectroscopy (EELS) and electron spectroscopic imaging (ESI)) revealed that chromium uptake was decreased when *Micrasterias* cells were pre-treated with iron, which resulted in no longer detectable intracellular chromium accumulations. Zinc rescued the detrimental effects of chromium on net-photosynthesis, respiration rates and electron transport in PS II. Calcium and gadolinium were able to almost completely compensate the inhibiting effects of lead and cadmium on cell morphogenesis after mitosis, respectively. These results indicate that cadmium is taken up by calcium and iron transporters, whereas chromium appears to enter the algae cells via iron and zinc carriers. It was shown that lead is not taken up into *Micrasterias* at all but exerts its adverse effects on cell growth by substituting cell wall bound calcium. The antioxidants salicylic acid, ascorbic acid and glutathione were not able to ameliorate any of the investigated metal effects on the green alga *Micrasterias* when added to the culture medium.

## Introduction

Metals are necessary components of all ecosystems and occur naturally in the earth's crust (Pinto et al., 2003). They appear in a wide range of oxidative states and coordination numbers, influencing their chemical characteristics and thus their bioavailability and toxicity (Pinto et al., 2003, Verbruggen et al., 2009). Certain metals such as iron (Fe), copper (Cu) and zinc (Zn) are considered essential nutrients to plants and are needed for photosynthesis and as cofactors for many enzymes (e.g. Kovacik et al., 2010, Shanmugam et al., 2011). Plants take up essential elements from their surroundings, but they are also able to accumulate elements, which have no known biological function, such as heavy metals like cadmium (Cd), chromium (Cr) or lead (Pb) (Mendoza-Cozatl and Moreno-Sanchez, 2005, Peralta-Videa et al., 2009). These nonessential metals are able to enter plant cells via metal transporters and carriers for the uptake of essential metals (Clemens, 2001, Shanker et al., 2005).

Aquatic environments are particularly exposed to increasing amounts of industrial and agricultural wastes (Kovacik et al., 2010). They may contain Cd, Cr and Pb which are toxic to most organisms at low concentration and have serious negative effects on plant growth, development and photosynthesis (di Toppi and Gabbrielli, 1999, Panda and Choudhury, 2005, Sacan et al., 2007, Peralta-Videa et al., 2009). Experimental amelioration of heavy metal effects by addition of antioxidants or essential ions provides insight into uptake and distribution mechanisms as well as on physiological and sub-structural targets of metals and increases our understanding on possibilities to limit damage to an aquatic ecosystem.

Antioxidants and certain essential micronutrients have the ability to inhibit heavy metal uptake, to contribute to detoxification or to decrease damage to plant cells. Salicylic acid (SA) is commonly known throughout the plant kingdom as regulator for physiological processes and as stress hormone during biotic and abiotic stress (Metraux, 2002, Belkhadi et al., 2010). Several studies demonstrate successful amelioration of heavy metal damage by SA (Guo et al., 2009, Belkhadi et al., 2010). The protective function of SA during heavy metal stress is not fully understood, but seems to mainly derive from its activity as antioxidant, scavenging reactive oxygen species (ROS) (Shah, 2003, Ahmad et al., 2011). The generation of ROS, either directly through Haber–Weiss reactions, or as a consequence of the metals toxicity, is the primary response of plants to heavy metal stress (Yadav, 2010). Other antioxidants such as glutathione (GSH) and ascorbic acid (AA) are also involved in the quenching of ROS, generally via the glutathione-ascorbate cycle (El-Naggar and El-Sheekh, 1998, Noctor et al., 2012, Bielen et al., 2013). GSH is a tripeptide and an indispensable small molecule to higher plants, with multiple functions in biosynthetic pathways, metal detoxification, antioxidant biochemistry and redox homeostasis (Zechmann et al., 2008, Noctor et al., 2012). Both AA and GSH were also found to be capable of diminishing heavy metal effects in the alga Chlorella (El-Naggar and El-Sheekh, 1998).

Among essential ions particularly Fe, Zn and calcium (Ca) may affect uptake and toxicity of heavy metals in plants and algae. Ca and Fe are able to reduce the uptake of Cd (Peralta-Videa et al., 2009). Ca has been shown only recently, to rescue Cd damage on photosynthesis and ultrastructure in the alga *Micrasterias* (Andosch et al., 2012) but was also found to have ameliorating functions on Cd effects in higher plants (Choi et al., 2001, Suzuki, 2005, Wan et al., 2011). Ca also has a positive effect on cell number and size of Pb treated protonema cells and reduced typical cell malformations found under Pb influence (Krzeslowska et al., 2004). Fe and Zn have been reported to ameliorate toxic effects and uptake of Cr in plant cells (Mallick et al., 2010, Branzini et al., 2012). All these ameliorating effects seem to arise mainly from chemical similarities of essential and toxic ions and their competition for carrier uptake into the plant cell (di Toppi and Gabbrielli, 1999, Shanker et al., 2005). This is also supported by experiments with gadolinium (Gd), a well-known Ca-channel blocker which diminishes Cd uptake, suggesting a rescue mechanism via Cd–Ca exchange (Hinkle et al., 1987).

In the present study we investigate ameliorating effects of signaling molecules, antioxidants and essential ions (AS, GSH, AA, Fe, Ca, Zn and Gd) on impact of the heavy metals Cd, Cr and Pb on the alga *Micrasterias denticulata* in order to obtain insight into heavy metal uptake mechanisms and intracellular targets. Previous publications have shown severe dose-dependent effects of different metals on growth, morphogenesis, photosynthetic and respiratory activity as well as on ultrastructure and function of organelles in the unicellular fresh-water alga *Micrasterias* (Volland et al., 2011, Volland et al., 2012, Andosch et al., 2012) which has been employed as a cell biological model since many years (e.g. Meindl, 1993, Oertel et al., 2004, Eder and Lütz-Meindl, 2008, Affenzeller et al., 2009; etc.).

By considering the fact that *Micrasterias* inhabits oligotrophic peat bog ponds and is adapted to extremely nutrient-depleted, low concentrated aquatic environments the hypotheses to be tested in the present study were the following: (1). Do metals such as Cd, Cr and Pb enter *Micrasterias* cells via natural transport systems, such as Ca, Fe or Zn channels like in higher plants? (2). Does addition of antioxidants or micronutrients, though unusually elevating the concentration of the algal environment, prevent damage by the metals? As *Micrasterias* belongs to a group of algae (Streptophyta) which are closest relatives of higher plants (Wodniok et al., 2011) the results of this study are not only relevant for our cell physiological understanding of heavy metal uptake but also in respect to an evolutionary point of view.

## Material and methods

### Chemicals

All chemicals were purchased from Sigma–Aldrich (Vienna, Austria), Alfa Aesar (Karlsruhe, Germany) or Carl Roth (Karlsruhe, Germany) unless stated differently.

### Cell cultures

*Micrasterias denticulata* cells were grown in liquid Desmidiacean culture medium (Schlösser, 1982) in Erlenmeyer flasks under semi-sterile conditions. The medium contained a substantial amount of soil extract providing good pH buffering properties. Cells were kept at 20 ± 1 °C at a photoperiod of 14 h light:10 h dark. Every 4–6 weeks the cells were sub-cultured. 3–4 week old cultures during exponential growth were used for experiments (for detailed culture conditions see Meindl and Lütz, 1996, Affenzeller et al., 2009).

### Light microscopy

To capture both, short-term metal effects on cell growth and morphogenesis and long-term impact on cell division rates, viability, ultrastructure and metabolic functions *Micrasterias* cells were exposed to short- and long-term incubations. For short-term treatments dividing cells (15–75 min after mitosis) were selected from cultures and were exposed to nutrient solutions containing different metals. For each metal the highest concentration was chosen which the cells were able to survive during a test series (see also our previous publications (Volland et al., 2011, Volland et al., 2012, Andosch et al., 2012). The following concentrations were used: 15 μM CdSO_4_ (Cd), 1 mM K_2_Cr_2_O_7_ (Cr) or 40 μM Pb(NO_3_)_2_ (Pb) respectively. For long-term treatments cell cultures were treated with 600 nM Cd,10 μM Cr and 5 μM Pb. After 4 h of incubation (short-term) or 21 days (long-term) of treatment, the effects on the cells were examined with a Univar light microscope (Reichert, Vienna, Austria) and documented with a Canon Powershot A620 camera (Tokyo, Japan).

The following agents were used for rescue experiments of heavy metals impact on *Micrasterias* cells: cultures were pre-treated with 20 μM salicylic acid C_7_H_6_O_3_ (SA), 20 μM L-ascorbic acid C_6_H_8_O_6_ (AA), 300 nM reduced l-glutathione C_10_H_17_N_3_O_6_S (GSH), 100 μM Fe-EDTA (Fe), 300 nM ZnSO_4_ (Zn) or 1 mM CaSO_4_ (Ca) for 1 week to allow acclimatization of the cells to the substances. Dividing cells were then collected and incubated for 4 h, or cultures were treated for 21 days with heavy metals and ameliorating substances simultaneously, before the effects were examined. For rescue treatments with gadolinium (Gd), cells from untreated cultures were exposed to 15 μM Cd and 40 μM Pb either together with 3 μM Gd or after 30 min pre-treatment with Gd. All used concentrations of rescue substances were previously determined to have no negative effect on cell development, morphology and photosynthesis when applied to *Micrasterias* cells alone. Heavy metals and rescue substances were added to the cell cultures only once at the given time points.

### Preparation methods for transmission electron microscopy and analytical TEM

*Micrasterias* cells after selected short- and long-term treatments (see above) were investigated in the TEM.

High pressure freeze fixation of treated cells and controls were done in a Leica EMPACT high-pressure freezer. Cryo-substitution was performed in a Leica EM AFS (Leica Mikrosysteme GmbH, Vienna, Austria) as described by Meindl et al. (1992) and Aichinger and Lütz-Meindl (2004). After freeze substitution cells were infiltrated and embedded in Agar low viscosity resin (Agar Scientific, Essex, U.K.) and polymerized for 16–24 h at 60 °C.

For structural analysis, ultrathin sections of 40–60 nm were cut on a Leica UC7 ultramicrotome (Leica Microsystems GmbH, Vienna, Austria) and were placed on formvar-coated copper grids for conventional imaging. For EELS measurements hexagonal narrow mesh copper grids were used. Sections were examined in a LEO 912 AB transmission electron microscope (Zeiss, Oberkochen, Germany) with in-column energy filter, operated with a LaB_6_ cathode and an acceleration voltage of 80 kV for conventional imaging and 120 kV for EELS. Micrographs and EELS were recorded with a slow scan dual speed CCD camera Sharpeye (Tröndle, Moorenweis, Germany), operated by iTEM software (Soft Image System, Münster, Germany).

### Intracellular metal localization via EELS and ESI

For the present study *Micrasterias* cells treated with 10 μM Cr plus 100 μM Fe, with 5 μM Pb for 21 days and with 40 μM Pb for 4 h respectively, were investigated.

For electron energy-loss spectroscopy (EELS) magnifications between ×25,000 and ×40,000 were chosen. The measurement area for EELS was defined by a 100 μm spectrometer entrance aperture and 5–7 integration cycles were taken per measurement. Cr and Fe were detected at the L-_2,3_ edge at an electron loss of 570 eV and 708 eV respectively. Illumination angles between 1 and 1.6 mrad, exposure times between 2 and 5 s and a spectrum magnification of 200× was used. For the detection of Pb via the M_4,5_ edge at a high energy loss of 2484 eV, a micrograph of the spectrum was captured as previously described by Zheng et al. (2012). The Pb M_4,5_ edge was then identified by superimposing an intensity profile over an image of the spectrum. Element maps (ESI) were taken with the three-window power-law method at a lower magnification of ×8,000.

### Measurement of photosynthetic activity

In order to determine the physiological status of treated *Micrasterias* cells, photosynthetic activity was measured by oxygen turnover (production/consumption) and by fast chlorophyll fluorescence (for method see Affenzeller et al., 2009).

### Photosynthetic oxygen measurements

For rescue experiments cells were pre-treated for 1 week with 20 μM AA, 20 μM SA, 300 nM GSH, 100 μM Fe and 300 nM Zn followed by a 21 days incubation in 600 nM Cd and 10 μM Cr. Additionally cell cultures treated only with 5 μM Pb for 21 days were measured. Roughly 2000 cells were used for each run with 3–4 light/dark cycles, which were repeated 3 times each. Oxygen turnover as indicator for photosynthetic activity was measured by a Hansatech (King’ Lynn, UK) polarographic oxygen electrode and μM oxygen/h/mg chlorophyll were determined. Illumination was set to 200 μM photons m^2^ s^−1^ to enable comparison with in earlier experiments (Andosch et al., 2012) and the temperature was kept constant at 20 °C. After each measurement an aliquot of the suspension was removed for a later determination of the total chlorophyll content by pigment extraction (Porra et al., 1989).

### Fast chlorophyll fluorescence

To assay the efficiency of photosystem II (PS II) activity a Handy PEA (Hansatech, King’ Lynn, UK) was used as described by Affenzeller et al. (2009). Five drops of the cell suspensions treated as described above were pipetted on pieces of filter paper and incubated in darkness for 20 min. A minimum of 7 parallel measurements was performed. During this incubation the cells were kept moist in the sample holders. PS II activity was expressed as *F*_v_/*F*_m_ (variable over maximum fluorescence as an introduced parameter) and the shape of the fluorescence induction curves was used to demonstrate the differences in fast energy conversion after the treatments in comparison to the controls, as has been described in detail by Strasser et al. (1995).

### Cell vitality

The percentage of living cells was determined by analyzing cell plasmolysis. Per treatment 50 cells were collected and the nutrient solution was substituted by 500 mM sorbitol (for method see Andosch et al., 2012). Cells not undergoing plasmolysis within 20 min sorbitol exposure were assumed dead and counted in a binocular (Nikon, Chiyoda-ku, Japan). Cell vitality-assays were carried out with 600 nM Cd, 600 nM Cd + 100 μM Fe, 600 nM Cd + 300 nM Zn and 10 μM Cr, 10 μM Cr + 100 μM Fe, 10 μM Cr + 300 nM Zn, 5 μM Pb after 21 days treatment. For rescue experiments cells were pre-treated for 1 week with the respective rescue agent prior to the experiment. All experiments were done in triplicate. 150 cells were analyzed in total. A Student’ *t*-test was done to determine statistical significance.

### Cell division rates

Dividing *Micrasterias* cells were selected and grown at culture conditions for 2 days in order to obtain interphase cells of the same age. Cell division rates of alga cells treated with 600 nM Cd + 100 μM Fe, 600 nM Cd + 300 nM Zn and 10 μM Cr + 100 μM Fe, 10 μM Cr + 300 nM Zn plus controls were examined over the course of 21 days. Division rates were compared with data of cells treated with 600 nM Cd and 10 μM Cr alone as previously published by Volland et al. (2012). The experimental setup was the same as above. All experiments were carried out in triplets starting with 10 interphase cells.

## Results

### Effects of Cd, Cr and Pb on *Micrasterias*

**Fig. 1:**
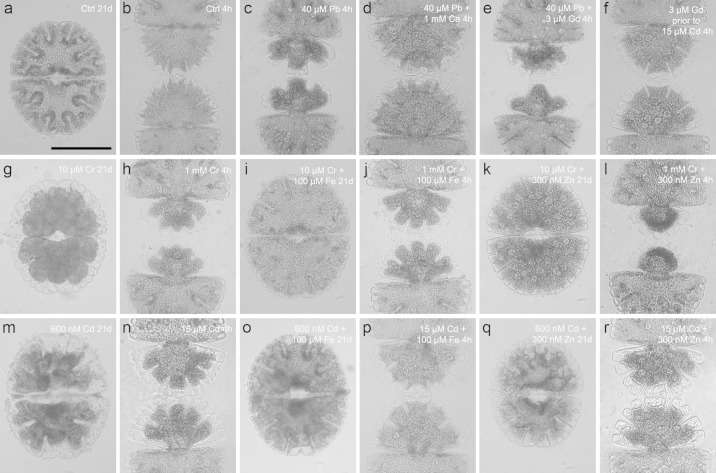
*Micrasterias* control cell in interphase (a) and fully developed approximately 5 h after mitosis (b); *Micrasterias* cells after various metals treatments (c–r): 40 μM Pb 4 h (c), 40 μM Pb + 1 mM Ca 4 h (d), 40 μM Pb + 3 μM Gd 4 h (e), 3 μM Gd (30 min) prior to 15 μM Cd 4 h (f), 10 μM Cr, 21 days (g), 1 mM Cr 4 h (h), 10 μM Cr + 100 μM Fe-EDTA, 21 days (i), 1 mM Cr + 100 μM Fe-EDTA 4 h (j), 10 μM Cr + 300 nM Zn, 21 days (k), 1 mM Cr + 300 nM Zn 4 h (l), 600 nM Cd, 21 days (m), 15 μM Cd 4 h (n), 600 nM Cd + 100 μM Fe-EDTA, 21 days (o), 15 μM Cd + 100 μM Fe-EDTA 4 h (p), 600 nM Cd + 300 nM Zn, 21 days (q), 15 μM Cd + 300 nM Zn 4 h (r); Scale bar 100 μm.

**Fig. 2:**
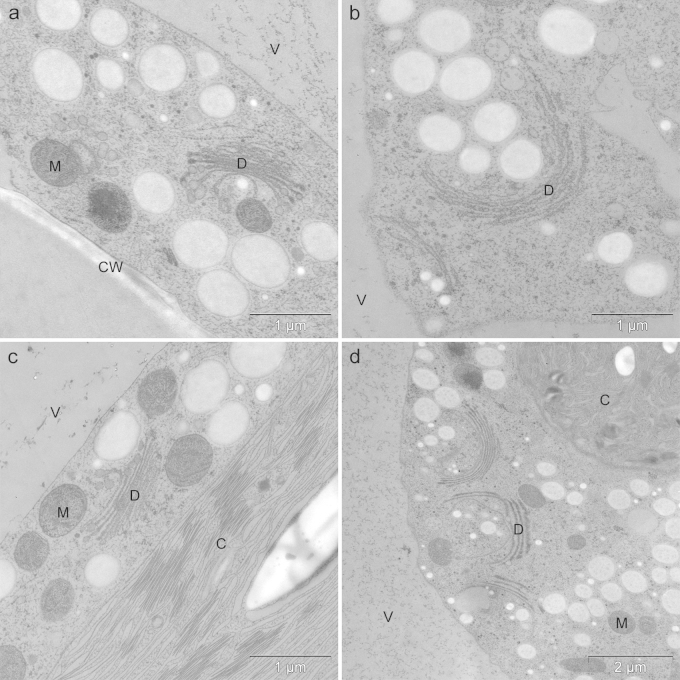
TEM micrographs of *Micrasterias* from control cells (a and b) and lead treated cells (c and d). Control 4 h (a) and 21 d (b) after mitosis. Short-term treatment (4 h) with 40 μM Pb (c) and long-term treatment (21 d) with 5 μM Pb. No ultrastructural differences between controls and treated cells. C chloroplast, CW cell wall, D dictyosome, M mitochondrium, V vacuole.

**Fig. 4:**
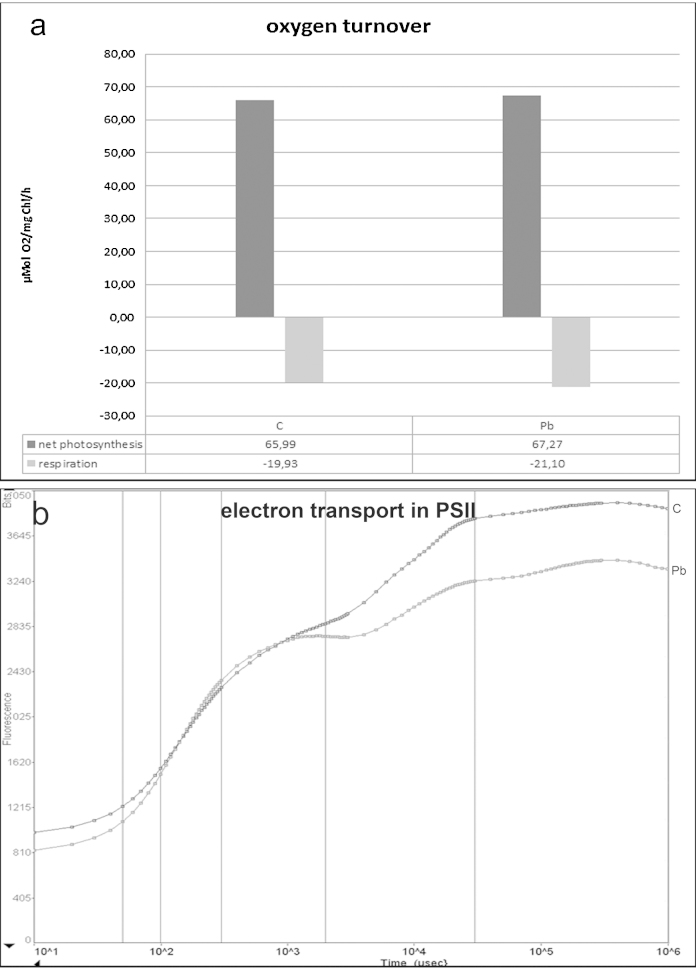
Oxygen turnover (a) and fast electron transport kinetics in PS II (b) after incubation with 5 μM Pb for 21 days.

40 μM Pb disturbed development of young half-cells in short-term treatments and occasionally led to cell death by bursting. The number of lobes was decreased and the tips were abnormally rounded (Fig. 1c) when compared to untreated controls (Fig. 1a and b). The polar lobe was often reduced, the lateral lobes were enlarged and the younger half-cells showed deeper indentations than controls. Nevertheless, the basal symmetry of the cell pattern was maintained. Cell vitality was reduced to 76.37% by exposure to 5 μM Pb for 21 days (Table 1). Interestingly, electron microscopic investigations did not indicate any ultrastructural changes of Pb short- and long-term treated cells (Fig. 2c and d) when compared to controls (Fig. 2a and b). Pb could not be detected by EELS (data not shown). The considerable energy loss of the Pb M_4,5_ edge at 2.484 eV and the arising unfavorable signal to noise ratio, however, may prevent detection of low Pb amounts. Photosynthetic oxygen turnover and PS II activity was not changed after 21 days treatment with 5 μM Pb (*F*_v_/*F*_m_ = 0.73) in comparison to controls (*F*_v_/*F*_m_ = 0.78; Fig. 4a and b).

**Table 1:** *T*-test: Cd and Cr tested against control, Cd treatment tested against Cd + Fe and Cd + Zn, Cr treatment tested against Cr + Fe and Cr + Zn; (paired *T*-test).

Treatment	Mean in %	SD	*T*-test	
Control	97.33	1.16		
600 nM Cd	55.33	8.08	0.0048	**
600 nM Cd + 100 μM Fe	96	2	0.008	**
600 nM Cd + 300 nM Zn	70.66	9.87	0.0145	*
10 μM Cr	75.33	11.55	0.0443	*
10 μM Cr + 100 μM Fe	96	0	0.0451	*
10 μM Cr + 300 nM Zn	90	6	0.0343	*
5 μM Pb	76.37	2.86	0.0051	**

* *p* < 0.05%.

** *p* < 0.01.

**Fig. 5:**
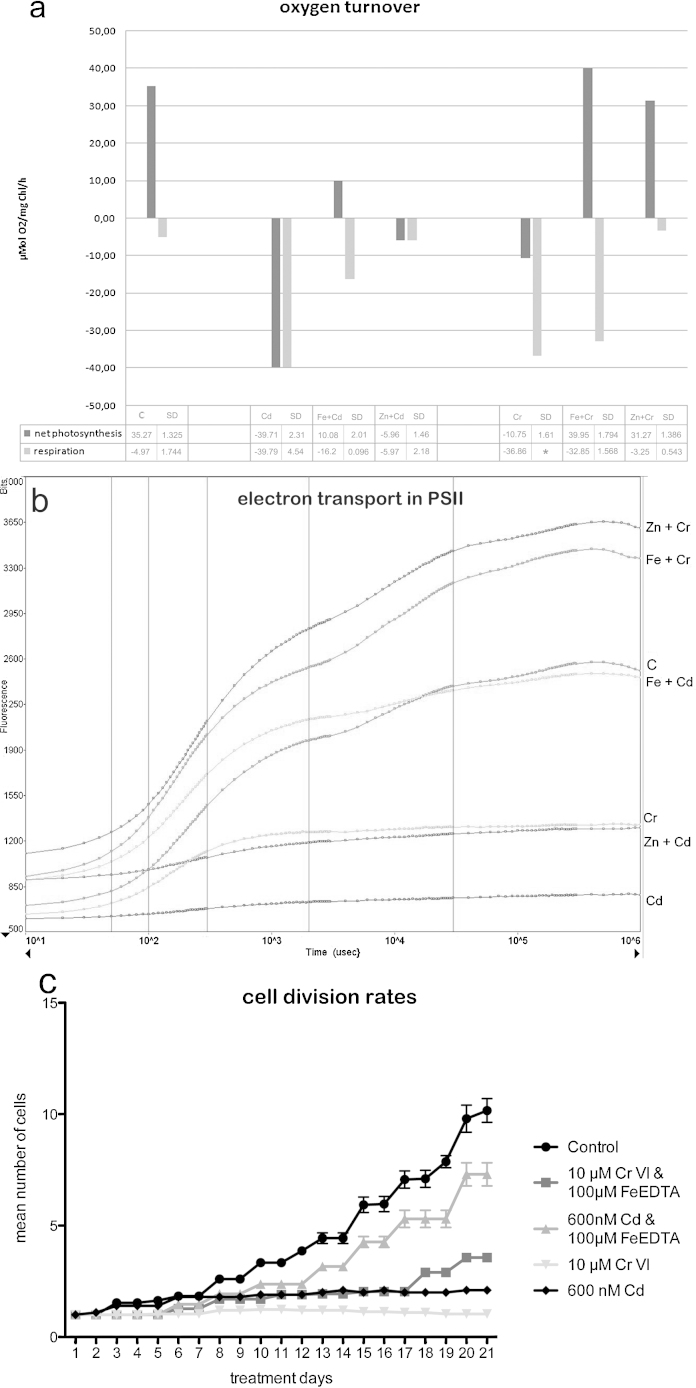
Oxygen turnover (a) and fast electron transport kinetics in PS II (b) after 21 days treatment time with 600 nM Cd and 10 μM Cr, with and without 100 μM Fe-EDTA and 300 nM Zn pre-treatment. Cell division rates over the course of 21 days during Cd and Cr treatment with and without Fe-EDTA pre-treatment (c).

In concentrations between 5 μM and 150 μM Cd, short-term treatments led to inhibition of cell growth and morphogenesis (Fig. 1n), the chloroplast appeared contracted and dramatic changes in cytoplasmic structures were observed (see also Volland et al., 2011, Andosch et al., 2012). Ultrastructural investigations revealed that the cells were strongly vacuolated, dictyosomes were disintegrated and autophagosomes appeared (Andosch et al., 2012). In *Micrasterias* cells treated with 600 nM Cd for 21 days, cell division rates were almost completely inhibited (Fig. 5c) and cell vitality dropped to 55.3% of the control level (Table 1). Cells were vacuolated and the chloroplast contracted (Fig. 1m). Net-photosynthesis reached negative values, respiration was increased and the electron transport in photosystem II was inhibited, as the flattened fluorescence induction curves (*F*_v_/*F*_m_ = 0.22) in comparison to the control show (*F*_v_/*F*_m_ = 0.75; Fig. 5a and b).

Cr in its hexavalent form was also found to inhibit cell growth and morphogenesis in short-term treated *Micrasterias* cells (Fig. 1h; see also Volland et al., 2012). Further, cell division rates were stagnant during long-term treatment (Fig. 5c) and the chloroplast appeared dark and contracted while lobe tips were vacuolated (Fig. 1g). Net-photosynthesis reached negative levels and the electron transport kinetics is disturbed (*F*_v_/*F*_m_ = 0.54; Fig. 5a and b). Cell vitality was reduced to 75.33% of that of controls (Table 1). Depositions of Cr in a compound with Fe and O were detected via EELS and ESI in specific, bag-like structures in the inner side of the cell wall during a recent study (Volland et al., 2012). Additionally, ultrastructural alterations were observed in the chloroplast and secretory activity was inhibited.

### Zn ameliorates the effects of Cd and Cr

Pre-treatment with 300 nM Zn could not rescue Cd or Cr effects on growth and development in short-term treated cells (Fig. 1l and r). In long-term treatments however, Zn pre-treatment affected the general appearance of Cr treated cells positively. Lobe tips were barely vacuolated and the chloroplast was not condensed (Fig. 1k). Net-photosynthesis was rescued to a sufficient level for cells to survive and respiration rates which went up during Cr treatment alone were drastically reduced by the pre-incubation with Zn (Fig. 5a). On the other hand, pre-treatment with Zn could not ameliorate effects of Cd treatment on general cell appearance (Fig. 1q) or on electron transport in PS II (*F*_v_/*F*_m_ = 0.31; Fig. 5b). Cd treated cells had no functioning electron transport, while 21 days Cr treated cells only showed the first hump of the typical “O-J-I-P” curve (Fig. 5b). These findings corroborate the effects previously found by Volland et al. (2012). Cr treated algae cells pre-treated with Zn showed a re-established electron transport chain. The fast kinetics of the electron transport in PS II was rescued although the basic fluorescence (*F*_0_) was elevated (Fm/Fv = 0.71; Fig. 5b). Further, Zn had no ameliorating effect on the cell division rates of Cd and Cr treated cells (data not shown), but was able the improve cell vitality from 55.3% to 70.66% in the Cd treatment and from 75.3% to 90% in the Cr treated cell cultures (Table 1).

### Fe ameliorates the effects of Cd and Cr

**Fig. 3:**
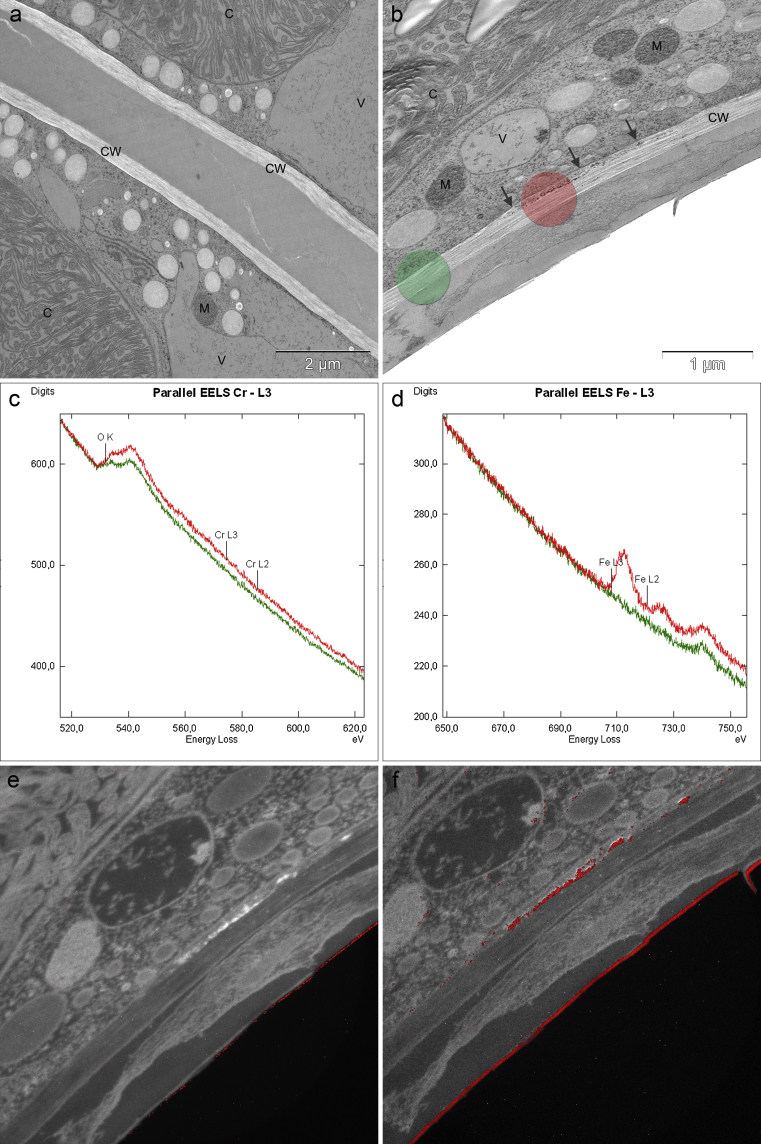
Detail of *Micrasterias* control cell (a) and ultrastructure, EELS and ESI of 10 μM Cr treated cell after 21 days pre-treatment with 100 μM Fe-EDTA (b–f). Arrow points at Cr induced depositions. Areas of EELS measurements indicated: green area without deposition, red area with deposition (b). EELS measurements of Cr-L_2,3_ edge (c), Fe-L_2,3_ edge (d). ESI overlay image indicating Cr distribution (e) and Fe distribution (f) in red. C chloroplast, CW cell wall, M mitochondrium, V vacuole.

Pre-treatment with 100 μM Fe was able to diminish the inhibiting effects of Cd and Cr on the cell development during short-term treatment and the younger semi-cells were able to develop further than after Cd and Cr treatment alone (Fig. 1j and p). The effects were even more distinct after long-term treatment (Fig. 1i and o), where the Cd and Cr exposed cells pre-treated with Fe-EDTA did not or barely differ from the appearance of the control cells (Fig. 1a). Ultrastructural investigations showed that Fe did not improve the impact of Cr in short-term treatments (data shown in Volland et al., 2012), when compared to the ultrastructure of cells treated with Cr alone. Fe pre-treated long-term Cr exposed cells, however, led to a decreased deposition of electron dense material along the inner side of the cell wall (Fig. 3b) in comparison to Cr treatment alone, where pronounced bag-like depositions appeared (data shown in Volland et al., 2012). Interestingly, in these depositions (measurement areas indicated in Fig. 3b) only O and Fe were measured at the O–K and the L-_2,3_ edge (Fig. 3c and d) and ESI (Fig. 3f). Cr could no longer be detected via EELS (Fig. 3c) or ESI as indicated by the element distribution map of Cr (Fig. 3e). Pre-treatment with Fe was further able to improve net photosynthesis of Cd and Cr treatment, but was not able to diminish respiration down to control levels (Fig. 5a). The inhibited electron transport in photosystem II was also rescued by the pre-treatment of Fe in Cd (*F*_v_/*F*_m_ = 0.65) and Cr (*F*_v_/*F*_m_ = 0.73) exposed cells (Fig. 5b) and cell division rates were improved (Fig. 5c). Additionally Fe pre-treatment improved cell vitality from 55.3% to 96% in Cd treated cells and from 75.3% to 96% in Cr treated cell cultures (Table 1).

### Ca and Gd ameliorate the effects of Cd and Pb

Dividing *Micrasterias* cells that were treated simultaneously with 40 μM Pb and 1 mM Ca finished their development like controls and no shape alterations were observed (Fig. 1d). Only in some cases the lobe tips were slightly and abnormally rounded. A simultaneous exposure to 40 μM Pb and 3 μM Gd did not ameliorate Pb effects. Growth of young half-cells was inhibited and cell deformations were similar to those after treatments with Pb alone (Fig. 1e). Treatment with 3 μM Gd for 30 min before exposure to 15 μM CdSO_4_ for 4 h, however, ameliorated Cd effects (Fig. 1f). Development of the young half-cells was almost completed in contrast to 15 μM Cd treated cells (Fig. 1n). The chloroplast appeared normal and lobe tips did not seem vacuolated (Fig. 1f). These findings are in good agreement with the results of a previous study (Andosch et al., 2012) giving evidence for ameliorating effects of extracellular pre-treatment of Cd exposed *Micrasterias* cells with calcium

### SA, AA and GSH were not able to ameliorate metal effects

In short-term treated Cd cells pre-treated with 300 nM GSH, growth and differentiation was slightly improved when compared to cells treated with 15 μM Cd. AA and SA had no positive effects on growth and development in short-term Cd or Cr treated cells. In long-term metal treated cells SA, AA and GSH did also not exhibit any ameliorating effects. Further cell division rates of metal treated cells pre-treated with SA, AA and GSH did not improve compared to cell division rates from metal only treated cells. Cd and Cr treated cell vitality rates did not change significantly in combined treatment with SA, AA and GSH (data not shown). Additionally, ultrastructural analyses of Cd treated cells pre-treated with SA were performed, but SA could not ameliorate the damaging effects of Cd on ultrastructure in short- or long-term treatments (data not shown).

## Discussion

Our results show that depending on the nature of the heavy metal, the divalent ions Ca, Fe, Zn and Gd are able to ameliorate previously reported negative metal impact on cell development, growth, division rates, photosynthesis and ultrastructure of the bog alga *Micrasterias* which grows under extremely nutrient-depleted conditions.

Pre-treatment with Fe was able to diminish the inhibiting effects of Cd and Cr on *Micrasterias* which were reported in previous studies (Volland et al., 2011, Volland et al., 2012, Andosch et al., 2012). Cell morphogenesis, photosynthesis, cell division rates and the overall appearance of the chloroplast in the algae were improved distinctly by Fe after long-term treatments. Zn on the other hand, only improved negative effects on photosynthesis and the general appearance of *Micrasterias* cells after long-term Cr treatment, but did not positively influence any other aspects of Cr, nor Cd toxicity. The powerful ameliorating effects of Zn and Fe seem to mainly derive from their chemical similarity to Cd and Cr (Shanker et al., 2005, Verbruggen et al., 2009). Cd has frequently been found to be taken up via Fe, Zn and Ca transporters in higher plants (DalCorso et al., 2008), whereas Cr uptake is known to compete with Fe, S and P for carrier binding (Shanker et al., 2005). These findings agree with our study concerning the ameliorating effects of Fe on Cd and Cr toxicity in *Micrasterias*. On the contrary, our results suggest that Cd uptake is not mediated through Zn transporters as in higher plants. Instead, Cr seems to additionally enter the algae cell via Zn specific carriers. Increased availability of the essential micronutrients Fe and Zn might not only have led to a decrease of heavy metal uptake, by competing for carrier binding, but might also have protected essential enzymes and proteins by their increased abundance within *Micrasterias* cells. Displacement of functionally active ions like Fe and Zn from enzymes and other proteins by heavy metals has been reported as another important reason for metal toxicity (di Toppi and Gabbrielli, 1999, DalCorso et al., 2008).

Zn has been found to completely inhibit Cd uptake in *Euglena gracilis* (Mendoza-Cozatl and Moreno-Sanchez, 2005) but cannot influence Cd effects in *Micrasterias*. This suggests different uptake mechanisms for Cd in these two algae. In contrast, increased concentrations of Fe have been frequently found to lead to reduced Cd uptake and toxicity in higher plants (Liu et al., 2008, Peralta-Videa et al., 2009, Mallick et al., 2010) and were also able to ameliorate the negative impact of Cd and Cr in *Micrasterias.* This points toward Cd and Cr uptake via Fe transporters in both cases. Zn also rescued the effects of Cr on photosynthesis, respiration and the electron transport in PS II. Thus, aside from a likely decreased uptake of heavy metals by excess amounts of Fe and Zn, these micronutrients also appear to have a protective or restoring function on photosynthetic activity in *Micrasterias*. This is possibly due to the fact that some enzymes in photosynthesis are Zn and Fe dependent like for example RuBisCO (Ribulose-1,5-bisphosphate carboxylase oxygenase) which is stabilized by Zn and catalyses the first step of carbon fixation (Stiborová et al., 1987, Curie and Briat, 2003).

The ultrastructure of Cr treated *Micrasterias* cells pre-treated with Fe only revealed ameliorating effects after long-term treatment. The most striking effect found was that dark granular precipitation contained in vesicle-like accumulations on the inner side of the cell wall after Cr treatment alone were reduced or disappeared completely when cells were pre-treated with Fe. Particular bag-like structures forming during Cr treatment alone (Volland et al., 2012) could no longer be observed after Fe pre-treatment. As identified by EELS and ESI, Cr exposed cells contained precipitations of Cr, Fe and O (Volland et al., 2012). In cells that have been exposed to Fe prior to Cr treatment these precipitations contained only Fe and O, whereas Cr could no longer be measured, indicating that less or no Cr was taken up in *Micrasterias* during combined metal treatment. This again confirmed our previous finding that a substantial amount of Cr enters the green algal cell through Fe transporters.

Pb causes shape alterations and cell death in developing *Micrasterias* cells but electron microscopic studies could not identify any structural changes in the cytoplasm, suggesting that Pb does not enter the cells. This is in agreement with the findings of Meindl and Röderer (1990), who discovered that PbCl_2_ induces cell death by bursting, but does not affect the ultrastructure of *Micrasterias*. However, these results do not correspond to studies in other plants where Pb has been frequently shown to be taken up into the cells and to influence intracellular components (e.g. Eun et al., 2000, Sacan et al., 2007, Kopittke et al., 2008, Jiang and Liu, 2010). Ca is able to compensate the impact of Pb on short-term treated *Micrasterias* cells, while Gd, a well-known Ca^2+^ channel blocker (Caldwell et al., 1998) has no ameliorative effects. This indicates that Ca^2+^ channels are not involved in the uptake and toxicity mechanisms of Pb in *Micrasterias*, even though they have been described as possible influx sites for Pb ions in other cells (Pourrut et al., 2011). We therefore suggest that disturbed cell shapes *in Micrasterias* after Pb exposure are probably due to an exchange of cell wall bound Ca^2+^ by Pb^2+^. Low-methyl-esterified pectins, components of the primary wall of *Micrasterias* are linked via Ca^2+^ ions (Eder and Lütz-Meindl, 2008), which can be replaced by di- or trivalent ions exhibiting a higher affinity for pectins, like for example Pb^2+^ (Krzeslowska, 2011). A subsequent change of cell wall plasticity during development could be responsible for the disturbed formation of the cell pattern, resulting in the bursting of cells. This competition of Ca^2+^ and Pb^2+^ for binding sites in the cell wall (Krzeslowska, 2011) could explain why Pb effects are diminished in the presence of high Ca concentrations in *Micrasterias* cells. Ameliorating effects of Ca on Pb toxicity have also been reported in higher plants like barley and *Festuca ovina* (Garland and Wilkins, 1981), in moss protonemata (Krzeslowska et al., 2004), maize, rye, tomato and mustard (Antosiewicz, 2005). Slaveykova and Wilkinson (2002) discovered that Pb uptake of the green alga *Chlorella vulgaris* was decreased in the presence of elevated Ca concentrations. There seems to be a connection between Pb effects and Ca throughout the plant kingdom, whereas the different underlying mechanisms are still not fully understood.

Previous experiments showed that the effects of 150 μM Cd on short-term treated *Micrasterias* cells were reversible upon pre-treatment with 2 mM CaSO_4_. Especially the detrimental Cd effects on photosynthesis, promotion of autophagy and in parts also the negative degradative effects on dictyosomal ultrastructure were rescued (Andosch et al., 2012). Cd has been reported to have a primary impact on Ca homeostasis, since it is able to displace Ca from binding sites and further shows a strong interference with the movements of K^+^ and Ca^2+^ in the cells (di Toppi and Gabbrielli, 1999). In the present study the Ca^2+^ channel blocker Gd was able to almost completely reverse the inhibiting effects of 15 μM Cd on developing *Micrasterias* cells. This indicates that Cd ions are at least partly taken up via Ca^2+^ channels, in contrast to Pb ions (as discussed above). Uptake mechanisms of Cd can differ in plant cells but have been reported to be partly mediated through Ca^2+^ channels (Mendoza-Cozatl and Moreno-Sanchez, 2005). Our study provides first evidence of a decreased uptake of Cd in the presence of Gd in plant cells, as to our knowledge the latter has so far only been employed in animal cells in this respect (Hinkle et al., 1987).

Rescue experiments by extracellular application of SA, AA and GSH were not successful concerning any aspect of Cd or Cr toxicity in *Micrasterias*, only GSH was able to slightly improve cell morphogenesis under Cd stress. To explain why these antioxidants were not able to significantly ameliorate heavy metal effects in *Micrasterias* is difficult. Heavy metals, especially Cr, are known to induce increased ROS production in general (Pandey et al., 2009) and also in *Micrasterias* (Volland et al., 2012) and a strong antioxidative defence and strong redox homeostasis has been linked to metal tolerance (Sharma and Dietz, 2008). GSH and ascorbate accumulate up to mM concentrations in stressed plant cells and play a crucial role in the defence against oxidative damage caused by ROS (Noctor et al., 2012). In the defence against heavy metal effects GSH has another important part as metal ligand and precursor for phytochelatins, commonly found during Cd detoxification in plants (Cobbett, 2000). In *Micrasterias* the phytochelatins PC_2–4_ have been identified after Cd exposure by means of HPLC and UPLC–MS (Volland et al., 2013). Nevertheless AA and GSH had no effect on Cd or Cr toxicity in *Micrasterias* and were probably either altered before entering the algae cells or not taken up at all. SA, though known to alleviate Cd induced growth inhibition in different plants by enhancing the oxidative defence mechanisms (Guo et al., 2009), did not ameliorate any of the heavy metal effects in *Micrasterias.*

In this study we were able to show that adding essential cations like Fe, Zn and Ca to the nutrient solution was able to diminish the effects of the heavy metals like Cd, Cr and Pb on cell development and morphology, ultrastructure, cell division rates and photosynthesis in *Micrasterias*. Our results further revealed possible uptake mechanisms of the investigated metals: Cd seems to be taken up by Ca and Fe transporters, Cr enters the algae cells via Fe and Zn transporters and Pb is not taken up at all by *Micrasterias* but exerts its negative effects via physical changes of the cell wall. Externally added antioxidants SA, AA and GSH were not able to ameliorate heavy metal effects on the green alga *Micrasterias* at all.
